# Supplementary material for: Incidence and determinants of Implanon discontinuation: Findings from a prospective cohort study in three health zones in Kinshasa, DRC
Source: PLoS One. 2020 May 11;15(5):e0232582. doi: 10.1371/journal.pone.0232582 (PMC7213683; doi:10.1371/journal.pone.0232582)
Supplement: S1 Table — (DOCX) [file pone.0232582.s001.docx]

S1 Table : Cumulative discontinuation rate over time

|  | n | cumulative discontinuation | Discontinuation rate |
| --- | --- | --- | --- |
| 6 months | 415 | 23 | 5.54 |
| 12 months | 415 | 35 | 8.43 |
| 18 months | 415 | 42 | 10.12 |
| 24 months | 415 | 83 | 20.00 |
