# Supplementary material for: Incidence and determinants of Implanon discontinuation: Findings from a prospective cohort study in three health zones in Kinshasa, DRC
Source: PLoS One. 2020 May 11;15(5):e0232582. doi: 10.1371/journal.pone.0232582 (PMC7213683; doi:10.1371/journal.pone.0232582)
Supplement: S2 Table — (DOCX) [file pone.0232582.s002.docx]

S2 Table : Reasons for early discontinuation stratified by predictors

|  |  | **Side effects** | | | | Partner opposed | | Preferred other FP method | | Other person opposed | | Wanted to become pregnant | | Other | |
| --- | --- | --- | --- | --- | --- | --- | --- | --- | --- | --- | --- | --- | --- | --- | --- |
|  |  | all | | Heavy bleeding | |  |  |  |  |  |  |  |  |  |  |
|  | N | n | % | n | %^¥^ | n | % | n | % | n | % | n | % | n | % |
| **Residence** |  |  |  |  |  |  |  |  |  |  |  |  |  |  |  |
| Military area | 29 | 22 | 75,9 | 4 | 18,2 | 8 | 27,6 | 3 | 10,3 | 3 | 10,3 | 1 | 3,4 | 2 | 6,9 |
| Non military area | 54 | 38 | 70,4 | 14 | 36,8 | 8 | 14,8 | 5 | 9,3 | 3 | 5,6 | 5 | 9,3 | 4 | 7,4 |
| **Live births** |  |  |  |  |  |  |  |  |  |  |  |  |  |  |  |
| ≤ 2 | 54 | 35 | 64,8 | 7 | 20,0 | 12 | 22,2 | 4 | 7,4 | 5 | 9,3 | 5 | 9,3 | 5 | 9,3 |
| 3 or more | 29 | 25 | 86,2^*^ | 11 | 44,0^*^ | 4 | 13,8 | 4 | 13,8 | 1 | 3,4 | 1 | 3,4 | 1 | 3,4 |
| **History of FP** |  |  |  |  |  |  |  |  |  |  |  |  |  |  |  |
| Has used injectable or implant in the past | 2 | 2 | 100,0 | 1 | 50,0 | 1 | 50,0 | 1 | 50,0 | 0 | 0,0 | 0 | 0,0 | 0 | 0,0 |
| Has not used | 81 | 58 | 71,6 | 17 | 29,3 | 15 | 18,5 | 7 | 8,6 | 6 | 7,4 | 6 | 7,4 | 6 | 7,4 |
| ***Heavy bleeding*** |  |  |  |  |  |  |  |  |  |  |  |  |  |  |  |
| Yes | 39 | 31 | 79,5 | 17 | 54,8^***^ | 12 | 30,8^*^ | 5 | 12,8 | 2 | 5,1 | 3 | 7,7 | 2 | 5,1 |
| No | 44 | 29 | 65,9 | 1 | 3,4 | 4 | 9,1 | 3 | 6,8 | 4 | 9,1 | 3 | 6,8 | 4 | 9,1 |
| **Method Information Index** |  |  |  |  |  |  |  |  |  |  |  |  |  |  |  |
| < 3 | 49 | 37 | 75,5 | 14 | 37,8 | 9 | 18,4 | 3 | 6,1 | 5 | 10,2 | 5 | 10,2 | 3 | 6,1 |
| 3 | 34 | 23 | 67,6 | 4 | 17,4 | 7 | 20,6 | 5 | 14,7 | 1 | 2,9 | 1 | 2,9 | 3 | 8,8 |
| Overall | 83 | 60 | 72,3 | 18 | 30.0 | 16 | 19,3 | 8 | 9,6 | 6 | 7,2 | 6 | 7,2 | 6 | 7,2 |

¥: % of all declared side effects; *p<0.05 ; **p<0.01; ***p<0.001
