# Supplementary material for: Incidence and determinants of Implanon discontinuation: Findings from a prospective cohort study in three health zones in Kinshasa, DRC
Source: PLoS One. 2020 May 11;15(5):e0232582. doi: 10.1371/journal.pone.0232582 (PMC7213683; doi:10.1371/journal.pone.0232582)
Supplement: S3 Table — (DOCX) [file pone.0232582.s003.docx]

S3 Table : Site where the clients went to remove the device

| Site | n | % |
| --- | --- | --- |
| Health Center | 54 | 64,7 |
| Hospital or Clinic | 22 | 27,1 |
| Nurse résidences | 7 | 8,2 |
