# Supplementary material for: Incidence and determinants of Implanon discontinuation: Findings from a prospective cohort study in three health zones in Kinshasa, DRC
Source: PLoS One. 2020 May 11;15(5):e0232582. doi: 10.1371/journal.pone.0232582 (PMC7213683; doi:10.1371/journal.pone.0232582)
Supplement: Attachment — Submitted filename: Cover letter and response to reviewers.docx [file pone.0232582.s004.docx]

March 15^th^, 2020

**To:**	Catherine S. Todd
Academic Editor
PLOS ONE

**Re:**	Revised version manuscript PONE-D-19-25274 R1

Dear Editor,

Thank you very much for the opportunity to revise and resubmit our manuscript. The comments we received helped to improve our research.

Our revised submission includes the following documents: (1) the manuscript with changes tracked, (2) a clean revised manuscript including tables and (3) the rebuttal letter with our responses to each point raised by the editor and reviewers.

Below is our reply to the comments and suggestions of the reviewers on a point-by-point basis.

We would like to thank the referees for the valuable and constructive comments and suggestions. We are hoping that we have adequately dealt with the comments and suggestions, and that the revised version is to your satisfaction.

Looking forward to hearing from you.

Sincerely,

Dr. Pierre Zalagile Akilimali

Kinshasa School of Public Health

University of Kinshasa

Democratic Republic of Congo

**Comments from the reviewer are below in bold font, and our responses follow each comment in plain font**

**Reviewer #1:**

**Page 4 -perhaps use another word instead of "opted" rather use chose? to use Implanon.**

We agree and have updated accordingly.

**Can you comment on the experience of nursing students to provide the service. Did they provide a better service as they knew research would evaluate their service provision.**

We appreciate this comment. To address this issue, we have added the following text to the discussion section: “The authors acknowledge that the performance of the nursing students could have been influenced by their awareness of being evaluated. Yet this influence could operate in opposing directions. Whereas they could perform better under observation than otherwise, they might also performance less well from being nervous about the observation.”

**What is meant by providing services at community level outreach event? was it a campaign to specifically target contraceptive uptake or the Implant? what other methods were available ? other health services? Could they have got implanon at a local service nearby (for post insertion support)**

As part of their practical training, the students provided FP services at the community level: at outreach events (mini-campaigns) set up in a central location (e.g., near a referral health facility, beside a market), door-to-door, or from their own homes. Nursing students offered a range of five contraceptive methods: Implanon NXT, DMPA-SC, oral pills, condoms, and Cycle beads. Prospective clients often preferred these community services to fixed facilities in their neighborhoods, because they were less expensive and required less waiting time.

**You say it was not possible to interview all 702, so what was the convenience sample limited to? those who agreed to stay?**

In addition to “willingness to stay,” the respondent needed to provide consent for the interview. So, out of the 702 women who received Implanon NXT, 531 women aged 18 to 49 years old agreed to participate in the study and gave their informed consent to be interviewed at 6,12 and 24 months.

**Why were the three districts chosen? noting one was very different? was there a programme that wanted to ensure Implanon was made available to the women in these areas?**

The selection of zones took into account the proximity of the training institutes in which the students were trained. In addition, for logistical efficiency, we needed to limit that number of health zones to be covered and selected three with different characteristics to capture different types of clients (to increase the generalizability of the results).

**Did you collect data on who removed the implant? was it the same nurses and where did they go?**

Nursing students received training in the removal of Implanon NXT in the 4th year of nursing school. At the large majority of outreach events, a more experienced nurse handled the removal of implants (not the nursing students), and often the client was referred to the health facility near the outreach site for the removal. Our data show that among the 83 clients who had had an implant removed, 65% went to the Health center, 27% to the hospital and 8% in the resident of Nurse to remove the device.

**The methodology says women were interviewed on the phone? on page 5 but a few sentences later it says women were asked to appear ? at a central community location. these two statements contradict each other. If many women lived in a military camp why did the study not ask for permission to interview them at the camp or did they also have to go to a community venue? again in analytic methods it says data was collected on a mobile phone?**

During this study, trained interviewers used smart phone technology to collect data during a face-to-face interview. The use of mobile technology for data collection allowed interviewers to automatically upload data to a secure electronic server instead of having to code and enter data manually. Data was collected using interview face to face using mobile phone as tools ( SurveyCTO). These were not phone interviews. However women were re-contacted by phone to schedule the follow-up interviews.

**Did women get reimbursed for participation?**

Participants received U.S. $5.00 as reimbursement for transportation costs.

**Could you have tracked the LTFP via clinics in case they had the implant removed?**

Unfortunately, this is not possible. The client record system at these clinics would not have this type of information, nor is it clear that women using the community service would ever have registered with the nearby clinic.

**The woman in military camps - were they working or just living with men employed by the military? if working perhaps the heavy bleeding impacted on their work or the camps had poor facilities? if they lack access to health facilities how did they get their implants removed? and if they all received equal counselling on a variety of methods why would they have less knowledge than other woman. Is it that the camps provide no Health facilities to their staff?**

Most of the participants from Military camps were the wives of military personnel. Military camps are closed settings in which the health structures are less likely to receive technical and financial support from development partners. Also, results from a previous survey show that women in military camps have lower levels of knowledge and use of contraception than do their civilian counterparts. Because one of these development partners has begun supporting the military camp health facilities in recent years, Implant users have access to trained personnel who can remove implants.

**Later in the conclusion it says the study was conducted in a community setting? rather enrollment was conducted in a community setting but interviews were on the phone? not in the community? see earlier comments.**

The study was conducted in a community setting and participants were interviewed face to face using a questionnaire programmed on a mobile phone. During this study, trained interviewers used smart phone technology to collect data. The use of mobile technology for data collection allowed interviewers to automatically upload data to a secure electronic server instead of having to code and enter data manually. Data was collected during face to face interviews using mobile phone as tools ( SurveyCTO). This was not a phone survey.
