# Supplementary material for: Incidence and determinants of Implanon discontinuation: Findings from a prospective cohort study in three health zones in Kinshasa, DRC
Source: PLoS One. 2020 May 11;15(5):e0232582. doi: 10.1371/journal.pone.0232582 (PMC7213683; doi:10.1371/journal.pone.0232582)
Supplement: Attachment — Submitted filename: Cover letter and response to reviewers.docx [file pone.0232582.s005.docx]

April 14^th^, 2020

**To:**	Catherine S. Todd
Academic Editor
PLOS ONE

**Re:**	Revised version manuscript PONE-D-19-25274 R2

Dear Editor,

Thank you very much for the opportunity to revise and resubmit our manuscript. The comments we received helped to improve our research.

Our revised submission includes the following documents: (1) the manuscript with changes tracked, (2) a clean revised manuscript including tables and (3) the rebuttal letter with our responses to each point raised by the editor and reviewers.

Below is our reply to the comments and suggestions of the reviewers on a point-by-point basis.

We would like to thank the referees for the valuable and constructive comments and suggestions. We are hoping that we have adequately dealt with the comments and suggestions, and that the revised version is to your satisfaction.

Looking forward to hearing from you.

Sincerely,

Dr. Pierre Zalagile Akilimali

Kinshasa School of Public Health

University of Kinshasa

Democratic Republic of Congo

**Reviewer’s Comments**

**Please add the description of the participants' context as military wives to the manuscript, ideally in the Introduction section.**

Kokolo health zone consists of compounds that house military personnel and their families. Military compounds differs from other health zones in that they are a closed encampment within the city with their own health system, though often underfunded and affording less access to family planning than in the non-military population. We have added a statement about this on page 5 in Method section.

**You identify the program as novel but do not mention the circumstances of the context where family planning services are traditionally limited and women less aware of contraceptive options.**

**Please add the reimbursement of travel expenses to the Methods section - this is a required feature to report.**

We have added the following statement “Participants received U.S. $5.00 as reimbursement for transportation costs per visit.” to page 7 of the manuscript.
